# Epiretinal Membrane Surgery: Structural Retinal Changes Correlate with the Improvement of Visual Function

**DOI:** 10.3390/jcm10010090

**Published:** 2020-12-29

**Authors:** Andrea Cacciamani, Pamela Cosimi, Guido Ripandelli, Marta Di Nicola, Fabio Scarinci

**Affiliations:** 1 IRCCS Fondazione Bietti, Via Livenza 3/5, 00198 Rome, Italy; andrea_cacciamani@hotmail.com (A.C.); pamela.cosimi@fondazionebietti.it (P.C.); guido.ripandelli@fondazionebietti.it (G.R.); 2 Department of Medical, Oral and Biotechnological Sciences, “G. d’Annunzio” University, 66100 Chieti, Italy; marta.dinicola@unich.it

**Keywords:** optical coherence tomography, pars plana vitrectomy, visual acuity, single retinal layer, automated segmentation

## Abstract

Several parameters have been studied for identifying the visual outcomes after pars plana vitrectomy (PPV) for epiretinal membrane (ERM) peeling. This retrospective study aimed to analyze structural retinal changes with spectral domain-optical coherence tomography (SD-OCT) and their correlations with visual acuity improvement in patients with ERM undergoing PPV. Twenty-one pseudophakic eyes were enrolled in the study. Ophthalmic evaluations included best corrected visual acuity (BCVA) and retinal layer thickness measurements with SD-OCT. The segmentation of the retina was divided into four parts: the inner retinal layer (IRL), composed of an internal limiting membrane, retinal nerve fiber layer, ganglion cell layer, and inner plexiform layer; inner nuclear layer (INL); outer plexiform layer (OPL); and outer nuclear layer (ONL). Correlations between changes in retinal layer thicknesses and BCVA were explored over a 6 month follow-up period. The postoperative thickness decrease of the INL was significantly correlated with thickness changes in all other measured retinal layers (*p* < 0.001). Non-parametric linear regression showed that postoperative improvement in BCVA was associated with a postoperatively decreased thickness in the IRL (*p* = 0.021), INL (*p* = 0.039), and OPL (*p* = 0.021). In eyes undergoing PPV, postoperative thickness decreases of the IRL, INL, and OPL were correlated with visual acuity improvements. Re-compaction of these retinal layers after relieving ERM-induced traction may be an important factor in postoperative visual function improvement.

## 1. Introduction

Idiopathic epiretinal membrane (ERM) is a common macular disease characterized by an avascular fibrotic membrane that develops between the vitreous and the internal limiting membrane (ILM) in healthy eyes of the elderly [1]. Although the pathogenetic mechanism is still unclear, it has been suggested that a posterior vitreous detachment (PVD) can produce microbreaks in the retina, resulting in migration and proliferation of fibroblasts and glial cells on the inner retina surface [2]. Conversely, another theory proposed that an abnormal dehiscence during the PVD may result in vitreoschisis, leaving some part of the vitreous body connected to the macula area [3].

A previous study showed that the levels of TGF*β*2 and nerve growth factor in vitreous samples of patients with ERM were increased [4]. The authors suggested that TGF*β*2 might stimulate the differentiation of a specific type of glial cells into myofibroblasts, by inducing their contraction in the ERM, while nerve growth factor might be involved in the activation of intra- and intercellular signals related to the progression of ERM pathology [4].

Furthermore, a possible correlation between miRNAs and fibrotic phenomena that characterize patients diagnosed with macula hole (MH) and ERM has been suggested [5].

Traction translated from an ERM to the inner surface of the retina can lead to vision loss, metamorphopsia, and macropsia. While several parameters have been studied for identifying the ideal time for surgery [6,7,8,9,10,11], visual outcomes after pars plana vitrectomy (PPV) for ERM peeling are variable and difficult to predict.

Determining the best time to perform PPV in patients with ERM is dependent on two main factors: the morphology of the ERM and underlying retina and the presence of cataract preoperatively.

In addition, the development or progression of nuclear sclerosis is a common complication of successful standard 23-gauge or 25-gauge PPV over the follow-up period. This represents an important bias influencing the postoperative evaluation of functional outcomes. Indeed, micro-incision techniques vitrectomy, such as 27-gauge non-vitrectomizing vitreous surgery, a procedure aimed at removing ERM without removing the vitreous, have been studied in comparison with the standard 25-gauge vitrectomy in patients with ERM and have shown not inferior outcomes [12,13].

Advanced stages of the disease are associated with disruption of the photoreceptors, which can cause poor postoperative functional recovery [14,15]. On the other hand, while early intervention in patients with milder ERMs has been shown to be beneficial [16,17], the demand for surgery is often limited in these patients.

Today, the ocular assessment of patients affected by ERM is completed with spectral domain optical coherence tomography (SD-OCT). This imaging technique is considered an essential component of the preoperative evaluation and allows for quantification of structural changes to the macula and specific retinal layers [18,19,20].

This study aimed to correlate morphological retinal changes using SD-OCT with visual function in pseudophakic patients affected by ERM after PPV for ERM peeling over a six month follow-up period.

## 2. Materials and Methods

### 2.1. Study Participants

In this retrospective study, we selected pseudophakic patients (who had undergone cataract extraction at least 180 days prior to PPV) who underwent 25-gauge PPV for ERM and ILM peeling from January 2019 to November 2019 at the Department of Ophthalmology of the IRCCS Bietti Foundation, Rome, Italy. All enrolled patients were diagnosed with idiopathic ERM classified as stage 3 (according to the Govetto classification system [21] and duration <3 years) confirmed by ophthalmoscopy and SD-OCT.

The local Ethics Committee approved this study (ERMLAB01 N° 77/18/FB) and all subjects signed an informed consent form.

We excluded patients with prior PPV, retinal photocoagulation procedures or other ocular diseases, ocular axial length >26.00 mm, and presence or history of systemic disorders known to result in retinal diseases, such as diabetes.

A complete ophthalmologic examination at baseline and 1, 3, and 6 months postoperatively included evaluation of best corrected visual acuity (BCVA) using Early Treatment Diabetic Retinopathy Study (ETDRS) acuity charts, measurement of intraocular pressure, fundoscopy, and SD-OCT scan obtained with the Spectralis HRA-OCT imaging platform (Version 1.5.12.0; Heidelberg Engineering, Heidelberg, Germany).

### 2.2. Image Acquisition

Spectral domain OCT (Spectralis HRA-OCT, version 1.5.12.0; Heidelberg Engineering, Heidelberg, Germany) images were obtained using the horizontal SD-OCT cross-section (13 lines spaced 250 μm apart) and were obtained preoperatively and at 1, 3, and 6 months postoperatively.

**Figure 1:**
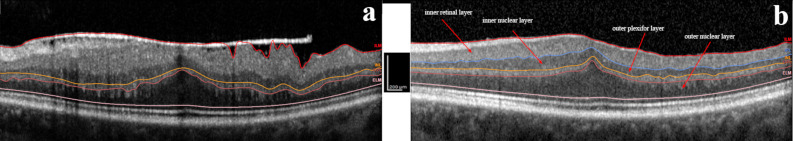
Spectral domain optical coherence tomography scan showing the epiretinal membrane traction causing foveal distortion and alterations of the inner and outer retinal structure of the four retinal layers (the layer between the red and blue lines corresponds to the inner retinal layer; the layer between the blue and yellow lines corresponds to the inner retinal layer; the layer between the yellow and salmon lines corresponds to the outer retinal layer; and the layer between the salmon and pink lines corresponds to the outer nuclear layer) obtained using automated segmentation algorithm preoperatively (**a**) and postoperatively (**b**). In this latter scan (**b**), there is the presence of a reduction in retinal layer thickness and the foveal contour is partially restored. (ILM, inner limiting membrane; IPL, inner plexiform layer; INL, inner nuclear layer; OPL, outer plexiform layer; ONL, outer nuclear layer).

Each OCT image was segmented using automatic built-in software. The segmentation of the retina was divided into four parts: the inner retinal layer (IRL), composed of an ILM, retinal nerve fiber layer, ganglion cell layer, inner plexiform layer; inner nuclear layer (INL); outer plexiform layer (OPL); and outer nuclear layer (ONL) (Figure 1).

Automated segmentation of all SD-OCT images was visually inspected by two graders (FS and PC), who were masked on patient identity, and correction was performed manually, if needed.

Since ERM traction caused an irregular alteration of the border of the retinal nerve fiber layer, ganglion cell layer, and inner plexiform layer in these patients, we measured all these layers as a single layer named the “inner retina” as aforementioned. The ILM and inner border of the INL were analyzed as the inner and outer borders of the “inner retina,” respectively [20].

The hyperreflective band of the ERM is occasionally partly detached from the ILM. When a hyporeflective space was present between the ERM and ILM, we placed the segmentation line along the inner margin of the ILM to exclude ERM [20] (Figure 1).

Two examiners (FS and PC), masked to all the clinical information, assessed the inner segment and outer segment (IS/OS) line, which was classified as intact, interrupted, or difficult to distinguish in the foveal scan. A continuous hyperreflective line corresponded to the intact IS/OS line. A photoreceptor abnormality was defined as focal alteration of the hyperreflective IS/OS junction.

### 2.3. Surgical Procedure

One surgeon (A.C.) performed the operative procedures using a 25-gauge standard 3-port PPV that included peeling of both the ERM and ILM. A PVD was created, if not already present, by applying high aspiration above the optic nerve and lifting the posterior hyaloid. After ERM peeling, the ILM was peeled using an intraocular dye composed of soluble lutein, brilliant blue, and trypan blue (Kemin Pharmaceutica Unipessoal, LTDA, Des Moines, IA) in all cases. A second stain with the intraocular dye was used to verify whether ILM peeling was complete.

### 2.4. Statistical Analysis

Qualitative variables were presented as frequencies and percentages. Continuous variables were examined for normal distribution using the Shapiro–Wilk’s test and described as mean and standard deviation (SD). A non-parametric Friedman test for repeated measures was performed to compare quantitative variables among different follow-up measures, and a relative post-hoc analysis was performed with Wilcoxon signed-ranks tests for pairwise comparisons.

The two-sided Kendall’s rank correlation coefficient Tau was computed to evaluate the relationship between retinal layer thicknesses pre- and postoperatively. Correlations were graphically depicted as correlation matrixes.

A non-parametric linear regression analysis was applied to evaluate the relationship between a change in BCVA from baseline to 6 months postoperatively and a change in retinal layer thickness over the same time period. This relationship was graphically depicted as a scatterplot with a linear regression line for each retinal layer. This analysis is a robust fitting method that is less sensitive to extreme observations (outliers).

Reliability between two graders for binary variables (status of the IS/OS junction) was assessed using Cohen’s kappa with 95% confidence intervals (CIs), with levels of agreement <0.40, 0.40–0.70, and >0.75 considered low, fair to good, and excellent, respectively.

False discovery rate correction (FDR) was applied to control the family-wise type I error rate, and a *q*-value < 0.05 was considered statistically significant.

All tests were two-sided, and the level of statistical significance was set at *p* < 0.05. The R software environment for statistical computing and graphics version 3.5.2 was used for the statistical analyses (R Foundation for Statistical Computing, Vienna, Austria. https://www.R-project.org/).

## 3. Results

Overall, 21 eyes of 21 patients (10 men and 11 women) underwent PPV for idiopathic ERM. The mean age of patients with ERM at the time of surgery was 68.0 ± 8.8 years. All eyes included were pseudophakic. All enrolled patients completed 6 months of follow-up. The mean duration of symptoms prior to surgery was 4.3 ± 0.8 years. No major adverse events occurred over the 6 months follow-up period (e.g., retinal detachment, retinal breaks, vitreous hemorrhages).

**Figure 2:**
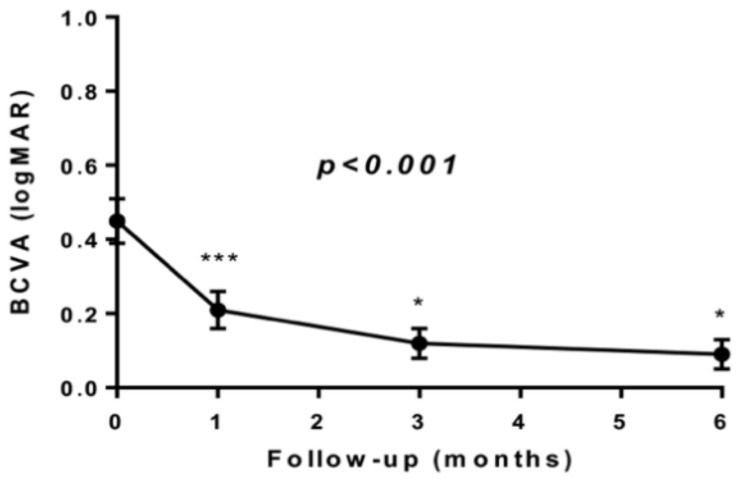
Mean and standard error of best corrected visual acuity (BCVA) during follow-up. The *p*-value reported in the figure is relative to the non-parametric Friedman test for repeated measures; * denotes a *p*-value < 0.05, *** denotes a *p*-value < 0.001 pairwise post-hoc analysis vs. previous measurement.

Compared to the pre-surgical baseline, there was a statistically significant improvement in BCVA at 6 months follow-up (0.45 ± 0.06 vs. 0.09 ± 0.04 logMAR, *p* < 0.001, Figure 2).

Compared to the pre-surgical baseline, the differences in the average thickness and central retinal thickness of all investigated retinal layers showed a statistically significant decrease in thickness following surgery (Table 1).

**Figure 3:**
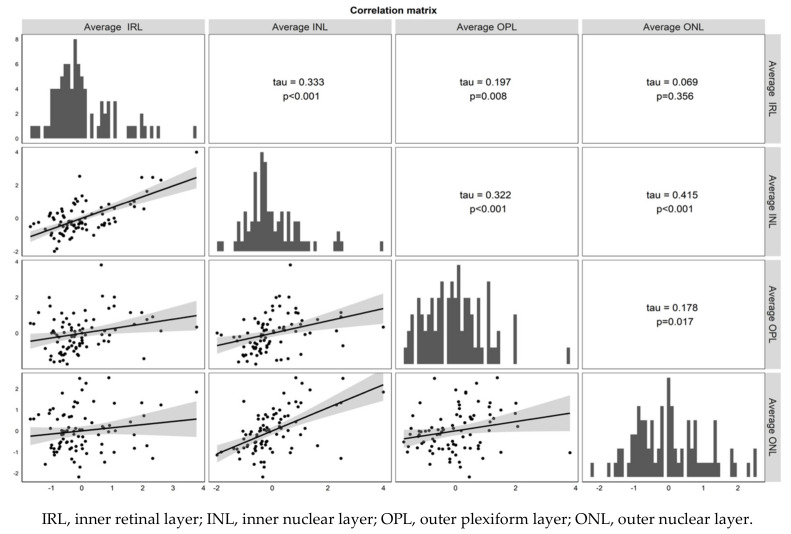
Correlation of average value of different layers of analyzed patients. Correlations were graphically depicted as correlation matrixes, using Tau correlation coefficients. Statistical significance was set at *p* < 0.05.

There was a statistically significant correlation between the postoperative decrease in INL thickness and postoperative decrease in all other retinal layers (Figure 3). 

**Figure 4:**
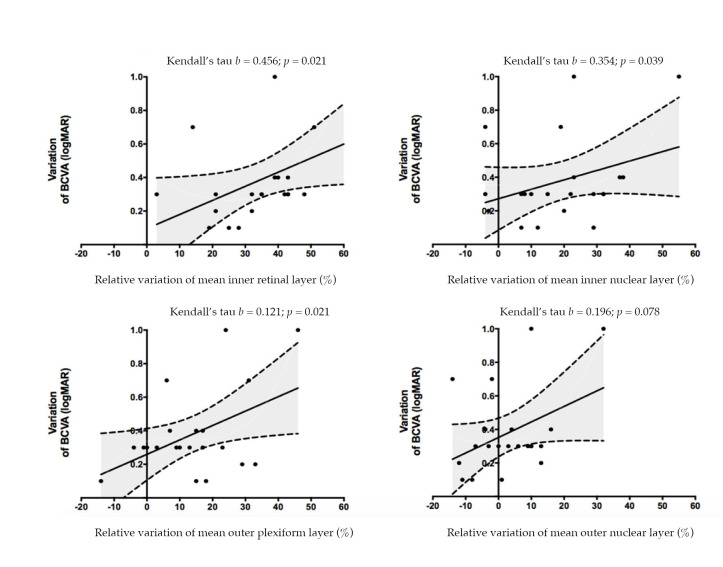
Non-parametric linear regression between variation of best corrected visual acuity and relative variation of layers thickness (expressed as average of different sector).

Postoperative improvement in BCVA, expressed as logMAR, following vitrectomy was correlated with a decrease in mean retinal thickness in the IRL (*p* = 0.021), INL (*p* = 0.039), and OPL (*p* = 0.021), whereas the postoperative change in BCVA did not have a statistically significant correlation with change in ONL thickness (Figure 4).

The foveal IS/OS junction was disrupted in 4 patients (19%), whereas it appeared normal in 17 patients (81%). The Cohen Kappa between the 2 examiners who evaluated the 21 patients was 0.911 (95% CI 0.905; 0.930).

## 4. Discussion

In this study, 25-gauge vitrectomy with ERM and ILM peeling was successfully performed in 21 patients with stage 3 ERMs and resulted in progressive improvement in BCVA during a 6 month follow-up period. Additionally, thickness measurements of the IRL, INL, OPL, and ONL steadily decreased over the 6 month postoperative period.

Looking at the functional and morphological findings together, we found that improvement in postoperative BCVA, compared to preoperative BCVA, was positively associated with postoperative thinning of the IRL (*p* = 0.021), INL (*p* = 0.039), and OPL (*p* = 0.021). On the contrary, we did not find any statistically significant correlation between changes in ONL thickness and visual acuity outcomes (Figure 4).

Looking at the structural OCT parameters, the correlation matrix analysis (Figure 3) showed that changes in the INL, expressed as mean, were correlated with the changes in all other layers (*p* < 0.001). In addition, a statistically significant correlation was found between the changes in the OPL and both the IRL and ONL (*p* = 0.014). Finally, the changes in the innermost and outermost retina layers (IRL and ONL, respectively) did not show any significant correlation (*p* = 0.435). These findings suggest that while thickness changes of different retinal layers can occur independently, changes in the INL have the strongest correlation with changes in other retinal layers following surgery for visually significant ERM.

Currently, it is still controversial as to which change and alteration of the intraretinal layer can participate in functional impairment in eyes affected by ERM pre- and after postoperatively.

With regard to this, Song et al. analyzed the thickness of several IRLs and their prognostic values and found that a thinner ganglion cell layer had a superior functional outcome [22].

Conversely, Kromer et al. found that greater postoperative visual acuity gain was associated with greater thickness of the retinal nerve fiber layer preoperatively [10]. However, in patients with ERM, the innermost retinal layers are not always well defined because of traction and it can be difficult to complete an accurate automated segmentation of all retinal layers even with state of the art SD-OCT platforms. Indeed, the differences in our findings, compared to prior studies [10,23], may be due to imaging performed at slightly different ERM stages and differences in SD-OCT platforms and software. In addition, unlike the inclusion criteria in the aforementioned studies, this study included only pseudophakic patients diagnosed with ERM classified at stage 3 according to the Govetto classification [21] and the “inner retinal layer” included the retinal nerve fiber layer, ganglion cell layer, and IPL in order to minimize any segmentation error. Differences in functional morphometric postoperative outcomes remained significant between the three ERM stages, with stage 2 and 4 ERMs at opposite ends. Patients with stage 4 were excluded with an aim to exclude eyes with chronic displacement of the inner retina that cause damage and deformations of photoreceptors and other retinal neural cells, compromising normal neural transmission [24]. These changes may not be fully reversible after ERM peel. Conversely, with the exclusion of patients with stage 2 ERM, we wanted to exclude eyes with good visual function [24], which might also influence the postoperative findings because of the ceiling effect.

Looking at the structural OCT findings (Figure 3), we observed that thinning of the INL was correlated with all the other layers.

More recently, changes in the INL have gained increasing interest in several retinal diseases [18,25,26] and it is fascinating to speculate the possible reason whereby changes of this layer and eventually its retinal remodeling might be important to the visual function in patients with ERM.

The retina is an advanced neuronal and vascular complex; specifically, the INL encompasses interconnections between amacrine, horizontal cells, and Müller cells. These cells lie at the opposite borders of the INL and are connected within the IPL and OPL, forming interconnections with the intermediate and deep capillary plexus [27]. Consequently, since Müller cells play an important role in facilitating the connection between vessels and neurons [28], both vascular and neuronal alterations might cause the development of retinal edema and neuronal cell death and contribute to retinal visual impairment in the advanced stages of ERM. Therefore, the postoperative thinning of the INL could facilitate the restoration of the neurovascular connections, allowing for better visual recovery [27].

Indeed, in a previous paper [20], we showed the importance of the biomechanical force primarily existing at the level of the ILM, which can reach via the Müller cell’s outer retinal layers, contributing to functional retinal dysfunction preoperatively.

INL thickness was also found to be important for the generation of metamorphopsia and as a biomarker of postoperative metamorphopsia in eyes with ERM [25,26].

The inner retina is largely nourished by the retinal vascular plexus, containing the superficial, intermediate, and deep capillary plexuses. Structural macular changes, such as those observed in this study following surgery for stage 3 ERM, may have an effect on local vascular function [29].

Indeed, vascular alterations in the macula area, including increased tortuosity and capillary obstruction, have been associated with reduced blood flow speeds in patients with ERM using fluorescein angiography [29].

More recently, using OCT angiography, Mastropasqua et al. showed that in patients with ERM, perfusion density, vessel length density, and vessel tortuosity of the superficial capillary plexus in the parafoveal region significantly improved within 6 months of postoperative follow-up [30].

Although it is still difficult to study the alteration of the deep capillary plexus in patients with ERM because of several artifacts, we cannot completely exclude the presence of vascular impairment of the deep capillary plexus preoperatively, which is located near the inner and outer borders of the INL in the parafoveal regions. Furthermore, Rommel et al. [31] showed significant choroidal sublayer perfusion changes postoperatively for ERM with an increase perfusion of choriocapillaris and a decrease perfusion of Sattler’s layer, by using OCT angiography. Additionally, preoperative Sattler’s layer perfusion was suggested as a useful predictive marker for functional results, since a significant correlation was found with postoperative visual function [31].

Based on the correlation between functional and structural results (Figure 4), we confirmed that BCVA improvement was associated with thinning of the IRL and INL, and we further highlighted a correlation with a thinning of the OPL.

This might be related to the fact that when tangential traction is released postoperatively, the cells regain orientation in the outer retinal layers, resulting in improved neural transmission.

Furthermore, from a vascular point of view, since the deep capillary plexus is fundamental for nourishing the high biochemical requirement of very specialized connections of the photoreceptors in the OPL [27,32], postoperative improvement of flow of this vascular plexus could partially increase oxygen tension at this level.

In our cohort of patients, we found that the foveal IS/OS junction was abnormal in only 4 eyes (19%); hence, we believe that the IS/OS condition had limited impact on our visual outcomes.

Limitations of this study include its retrospective design and the relatively small number of patients classified as stage 3, representing only a part of all patients who underwent surgery for ERM. Indeed, patients with more advanced stages were not included. Nevertheless, a longer follow-up is needed to confirm these preliminary findings. Finally, automated segmentation analysis does not include the evaluation of changes in the outer retina morphology, which have been shown to contribute to functional postoperative outcomes [33].

However, we would like to highlight the strengths of this study, which included only pseudophakic patients classified with the same stage of disease.

## 5. Conclusions

Based on these findings, we observed that the improvement of visual function in patients with stage 3 ERM was associated with thinning of the inner retinal layers as well as the OPL layer postoperatively. Failure to have significant postoperative thinning of these layers may be a possible reason for sub-optimal visual acuity outcomes after ERM peeling. Moreover, the neurovascular unit should be further analyzed with functional evaluation, including microperimetry and electro-functional studies.

## Figures and Tables

**Table 1:** Anatomical spectral domain optical coherence tomography parameters (parameter mean value ± standard deviation in micron) during the follow-up.

	Pre-Operative	1 Month	3 Months	6 Months	Relative Variation vs. Baseline	*p*-Value ^†^
IRL central	159.57 ± 46.90	111.33 ± 19.16 ***	102.29 ± 20.39 **	100.62 ± 17.41	−0.31 ± 0.25	<0.001
Average IRL	179.7 ± 32.1	130.2 ± 16.9 ***	120.4 ± 17.2 **	115.7 ± 16.8 **	−0.34 ± 0.13	<0.001
INL central	87.05 ± 26.19	73.14 ± 16.37 ***	65.57 ± 12.94 **	62.10 ± 13.87 **	−0.25 ± 0.19	<0.001
Average INL	70.8 ± 14.7	62.3 ± 10.42	58.3 ± 8.60	55.53 ± 8.07 *	−0.19 ± 0.15	0.014
OPL central	51.48 ± 10.78	43.86 ± 9.75 ***	41.14 ± 7.67 **	38.29 ± 6.40	−0.23 ± 0.15	<0.001
Average OPL	47.2 ± 8.0	43.0 ± 5.72 ***	41.5 ± 4.66 *	39.85 ± 5.92	−0.14 ± 0.13	<0.001
ONL central	128.10 ± 27.34	122.10 ± 23.22 *	118.86 ± 21.69 *	115.10 ± 19.27	−0.08 ± 0.14	0.005
Average ONL	91.2 ± 15.8	92.9 ± 14.8	90.8 ± 13.66 *	87.28 ± 12.32 **	−0.03 ± 0.11	0.043

IRL, inner retinal layer; INL, inner nuclear layer; OPL, outer plexiform layer; ONL, outer nuclear layer. ^†^ Non-parametric Friedman test for repeated measures * *p* < 0.05, ** *p* < 0.010, *** *p* < 0.001 pairwise post-hoc analysis vs. previous measurement. Bolded *p*-values are significant after false discovery rate correction.

## Data Availability

The data presented in this study are available on request from the corresponding author.
